# Local and traditional knowledge systems, resistance, and socioenvironmental justice

**DOI:** 10.1186/s13002-023-00641-0

**Published:** 2024-01-04

**Authors:** Natalia Hanazaki

**Affiliations:** Departamento de Ecologia E Zoologia, Centro de Ciências Biológicas, Universidade Federal de Santa Catarina, Campus Universitário S/N, Florianópolis, SC 88040-900 Brazil

**Keywords:** Decoloniality, Environmental justice, Indigenous peoples, Local communities

## Abstract

In this essay, for the debate series of Journal of Ethnobiology and Ethnomedicine, I argue against the oversimplified causal argument that the maintenance of local and traditional knowledge systems is related to less advantaged circumstances. This statement is based on a colonialist perspective of what a less advantageous circumstance is, which is being questioned by several authors. It also ignores the struggles and resistance of traditional knowledge holders and the urgent call for socioenvironmental justice. As an ethnobiologist, I argue that we must face this reality to build science with justice and inclusiveness.

## Introduction

My general position about the question, “*Are local knowledge systems still practiced mainly because of less-advantaged circumstances?*,” proposed for the debate series of Journal of Ethnobiology and Ethnomedicine, is against this statement on two general levels: first, what lies behind the idea of less-advantaged circumstances, and second, this statement ignores the struggles, resistance, and choices of local and traditional knowledge holders.

This statement is inspired by a proposition of a dilemma about the reasons why traditional ecological knowledge is maintained: not by the choice of TEK holders but by their “lack of choice underpinned by poverty and deprivation” [1]. In that sense, the postulated less-advantaged circumstances is directly related to poverty and deprivation. As a starting point, it is important to situate that here I am using a broad understanding of local and traditional ecological knowledge to refer to these knowledge systems as a cumulative body of knowledge, practices, and beliefs, which is adaptive, with intergenerational transmission, about the relationships between living beings and environment [2]. Despite the nuances between TEK and LEK (see, for example, [3, 4]), and regardless of the choice of one expression or other, both concepts share a central characteristic that this knowledge is not static. Being adaptive, it is constantly changing, absorbing novelties, and generating innovations [2]. Part of the motivations behind the abovementioned question on the relationship between local/traditional knowledge systems and less-advantaged circumstances arise from the modern epistemic division between academic knowledge and local/traditional knowledge systems [5]. The origin of this division was shaped by a colonialist perspective (see also [6] for this debate), which usually considers the former as mechanistic and the latter as more holistic [7] and creates artificial boundaries that marginalize local/traditional knowledge [7].

In a simplistic approach, it is easy to agree with the general idea that local and traditional knowledge systems remain alive mostly when associated with less-advantaged circumstances associated to poverty and deprivation, especially from the perspective of urban/industrial/developed countries contexts. However, the causal relationship between the maintenance of the practices underlying TEK and LEK and less-advantaged circumstances must not be assumed as the unique or primary reason for the maintenance of these knowledge systems. Pockets of local and traditional knowledge are frequently associated with rural groups and indigenous peoples who usually remained at the margins of mainstream economic growth without easy access to the "benefits of modernity." Usually, these less-advantaged circumstances are related to economic indicators of poverty. For example, the most prominent indicator of poverty is measured by poverty lines, which, although open to criticisms, are defined as a measure expressed in the amount of dollars per day, and used as the common standard of what “poverty” means. Since the purchasing power may vary depending on the country, several poverty lines can be used, for example, those varying in the upper limits from $3.20 and $5.50 per day, for national poverty lines typically found in lower- and upper-middle income economies, respectively [8], and the International Poverty Line of $2.15 [9]. These indicators help to analyze general scenarios, set goals for poverty reduction, and conclude, for example, that the percentage of people living below these standards has reduced [10]. In summary, poverty can be reduced to a monetary measure: a higher income is necessary for fewer people in poverty. However, useful for economic comparisons of indicators, this simplification ignores other essential measures of less-advantaged circumstances, such as inequality. Inequalities are not only driven and measured by income [11] but are influenced by various factors, including gender, age, origin, ethnicity, class, and religion.

## Other layers to understand economic disadvantages

All those measures are based on the current economic model whose foundations are colonialism and the associated genocides and structures of organized violence [12], which are the same structures that resulted in the current situation of marginalization of indigenous peoples and traditional communities [12, 13]. With this ruler for measuring poverty, there will inevitably be a congruence between TEK holders and poverty. This is why we need to analyze this congruence critically and why I am politically against this statement. To add to this debate, many other layers of "less-advantaged circumstances" can be added here, such as the access and safeguarding of territories, especially for indigenous and traditional people. Unsafe [14] and unsecured territories [15], displacement and trauma [16] also impose direct threats to local and traditional knowledge systems, including those of diasporic communities living in metropolitan centers, who sustain the continuity of their traditional knowledge and contradict the poverty assumption.

Under this perspective, the "less-advantaged circumstances" result from (and are defined by) a context in which traditional people historically did not had voice, participation, and–more importantly–their values considered and respected. These less-advantaged circumstances also result from the value systems that underlie the environmental conflicts affecting indigenous peoples and traditional communities. According to [17], traditional peoples contest the instrumental reduction of nature imposed by the global capitalist logic and the urban-industrial territoriality. This criticism is also addressed to debates about solutions for an environmental crisis that come from the dominant perspective yet ignore socioenvironmental justice, such as sustainability, in the indigenous perspective [18]; and Blue economy, in the perspectives of artisanal fishers [19]. Social injustices against traditional knowledge holders include disrespect, misrepresentation, invisibility, misunderstanding, economic and political vulnerability, unethical collaborations, rights violations, disconnection, uncontextualized education, and a lack of inclusivity [20]. According to [21], Neoliberalism impacts all socially marginalized groups; still, the disadvantages experienced by Indigenous people are worse due to the continued effects of colonialism, institutional racism, and intergenerational trauma. Hence, we need to face these challenges, which are a legacy of colonialism, in approaches focused on TEK, such as those from ethnobiology [6].

Thus, I understand that, as with any model, a stylized explanation such as the one proposed in [1] oversimplifies the reality and, in this sense, it is easy to agree with this extreme side of that schematic gradient, where the traditional and local ecological knowledge may be maintained because of the lack of choice underpinned by poverty or deprivation. The relative definitions of poverty vary according to the country and even within countries, with inequality effects deeply embedded in how the economic situation affects individuals, communities, and nations. However, even in extreme poverty, there are several layers of motivations to keep–or abandon–local ecological knowledge. As stated above, TEK and LEK are not static [2] and, as adaptive knowledge, are constantly changing to face new challenges and pressures. If one understands modernization as a challenge to be adapted to, TEK and LEK will remain alive to the extent that there is a cultural identity that makes sense to its holders. When TEK and LEK are lost, what is lost in the first place is this cultural identity, which is related to history, culture, and sense of place.

## Local and traditional knowledge system's resistance as a choice

Another delicate point that needs to be addressed is the simplistic explanations of how and why local and traditional knowledge systems persist over time also reflect a legacy of inadequate models of the evolution of human societies, from primitive nomadic and agricultural societies toward urban/industrial societies ([22], see also [23], present in several cultural anthropology books from the twentieth century, which biases the simplistic approaches toward a unidirectional cultural evolution. Thus, it has an implicit expectation that farmers' systems may fit into this trajectory, with living TEK as a representative of a romantic past, which is going into a future with lost TEK in subsistence or industrialized farming, along with changes in the environment from a pristine condition to a highly human-controlled ecosystem.

We can find several examples of how local knowledge systems thrive for reasons other than “less-advantaged circumstances.” This does not mean that poverty or deprivation were apart from the triggers of these choices. In agroecological systems, traditional knowledge plays an important role (e.g., [24, 25]), and in several places, the adoption of agroecology is a choice of resistance of indigenous and peasant movements [26] in response to the social and environmental problems of the prevailing industrial model of agricultural production [27]. At first glance, these practices can be criticized for being labor-intensive compared to other “modern” forms of agriculture, but even this concept can be questioned. The quality work associated with agroecological farming methods contributes to developing skills and capabilities related to greater self-determination and safeguarding the continuity of family farms [28].

When religious practices and beliefs are connected to local/traditional knowledge systems, the permanence of TEK and LEK is less dependent on the conventional economic indicators of advantaged or disadvantaged circumstances. In Brazil, for example, temples of religions from the African matrix, such as Candomblé are present all over the country (see, for example, [29–31]), even in urban areas with very different development indicators. In these religions, plants and ethnobotanical knowledge play a central role [29]. These places are spaces of resistance to practices, beliefs, and knowledge, exemplifying how traditional knowledge systems thrive even within highly urbanized contexts. For example, [32] discussed the adaptations of Candomblé and plant uses in New York city, within a context where Candomblé was introduced from Brazil.

The traditional knowledge systems related to religions of the African matrix can be used in complementarity with biomedicine therapeutic practices [29, 33]. Similarly, based on several examples from South America, [34] emphasize the role of local ecological knowledge in the adaptation, transformation, and resilience of ethnomedical systems. In these examples, treatments based on traditional ecological knowledge are not used as a last-resource option but as a choice for a complementary or alternative therapeutic path [35].

Moving toward urban areas, where local and traditional ecological knowledge could be expected as inexistent, we have several examples of the maintenance and generation of local knowledge, such as in a peri-urban area adjacent to Barcelona with a heavily anthropized mosaic of urban, industrial, agricultural, and conservation use [36]. Within urban centers, other examples of the resistance of local and traditional knowledge systems can be found in multicultural contexts. For example, for diasporic communities, when people migrate to a new context, they carry with them their ethnomedical practices and beliefs, and they modify their interactions with medicinal plants [37, 38]. Often, they also keep and modify traditional knowledge related to food plants and recipes, and the maintenance, loss, and adaptation of this knowledge can be observed in different degrees [39]. The major drivers for these changes in knowledge systems depend on the importance of keeping a given cultural identity and inclusivity and respect for biocultural diversity [39].

From the indigenous perspective of Krenak [18], the political dilemma that remains for indigenous peoples is still having to fight for the last strongholds where nature is prosperous, where they can meet their needs, and where each of these small societies can survive by themselves without creating an excessive dependence on the State. I recognize that many knowledge holders are experiencing poverty as a consequence of these multiple layers of social injustices; however, more than hostages of poverty traps, the traditional knowledge systems are strongly rooted in resistance and survival. More than neoliberal economic measures, LEK and TEK holders need social and environmental justice.

Finally, I recognize that there are situations where local knowledge systems are still practiced mainly as a last-resource option driven by poverty and lack of access to other alternatives. Examples of these situations are when marginalized people have extractive activities such as collecting plants as their last resort because they have no other choice to generate a minimum income. Often, these activities will depend on local knowledge, which may not be historically constructed but is generated as a necessity for the most basic survival. However, in line with [40], we should avoid unproductive debates based on polarized positions in ethnobiology: on the one hand, the extremes in a gradient are didactic ways to understand reality; on the other hand, they are excessively simplistic, and thus incomplete, to navigate in the complex world we are living today.

## Data Availability

Not applicable.

## Abbreviations

LEK: Local ecological knowledge
TEK: Traditional ecological knowledge
